# Middle ear osteoma causing progressive facial nerve weakness: a case report

**DOI:** 10.1186/1752-1947-8-310

**Published:** 2014-09-18

**Authors:** Kate Curtis, Manohar Bance, Michael Carter, Paul Hong

**Affiliations:** 1 IWK Health Centre, Department of Surgery, Dalhousie University, 5850/5980 University Avenue, PO Box 9700, Halifax, NS B3K 6R8, Canada; 2 Department of Pathology, Dalhousie University, Halifax, NS, Canada

**Keywords:** Facial nerve dysfunction, Middle ear lesions, Middle ear osteoma

## Abstract

**Introduction:**

Facial nerve weakness is most commonly due to Bell’s palsy or cerebrovascular accidents. Rarely, middle ear tumor presents with facial nerve dysfunction.

**Case presentation:**

We report a very unusual case of middle ear osteoma in a 49-year-old Caucasian woman causing progressive facial nerve deficit. A subtle middle ear lesion was observed on otoscopy and computed tomographic images demonstrated an osseous middle ear tumor. Complete surgical excision resulted in the partial recovery of facial nerve function.

**Conclusions:**

Facial nerve dysfunction is rarely caused by middle ear tumors. The weakness is typically due to a compressive effect on the middle ear portion of the facial nerve. Early recognition is crucial since removal of these lesions may lead to the recuperation of facial nerve function.

## Introduction

Tumors of the middle ear space are rare. Like middle ear effusions, they can cause conductive hearing loss and other otologic symptoms. Vascular lesions, such as glomus tumors, most commonly affect the middle ear [1]. Normal anatomic variants, such as dehiscent jugular bulb or high-riding carotid artery, may also invade the middle ear space.

Lesions arising from the temporal bone itself are rare [1,2]. Osteomas are the most widespread neoplasms of the temporal bone [3]. They tend to occur in the external auditory canal but can also occur in other parts of the temporal bone, such as the middle ear space [3,4], where the facial nerve is situated. Subsequently, middle ear lesions can cause facial nerve dysfunction. However, facial nerve weakness is usually caused by other pathologies, such as Bell’s palsy or central lesions.

Middle ear osteomas are rare benign tumors that may present with conductive hearing loss and tinnitus [5]. Facial nerve involvement is extremely rare but requires early recognition. In the present study, we report a case of middle ear osteoma causing progressive facial nerve paresis.

## Case presentation

**Figure 1:**
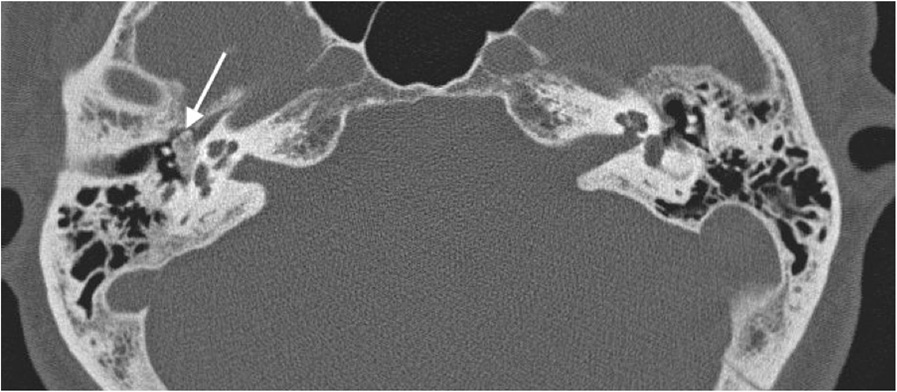
An axial computed tomographic image showing an osseous lesion in the right middle ear space (arrow).

**Figure 2:**
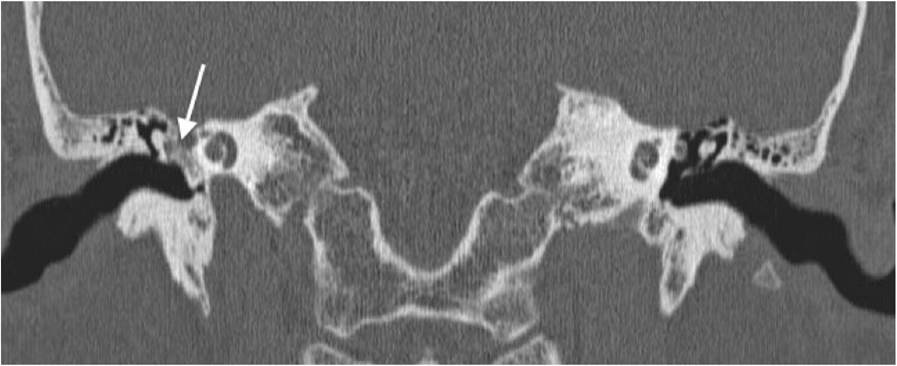
A coronal computed tomographic image showing an osseous lesion in the right middle ear space (arrow).

**Figure 3:**
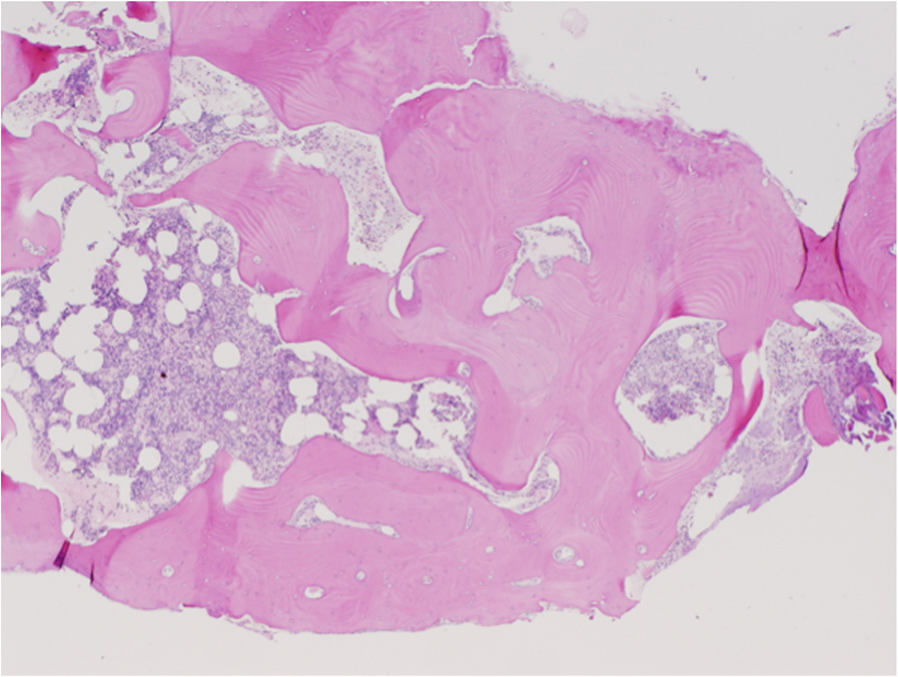
Histopathological section of middle ear osteoma showing fragments of lamellar bone with normal marrow elements (stained with hematoxylin and eosin).

**Figure 4:**
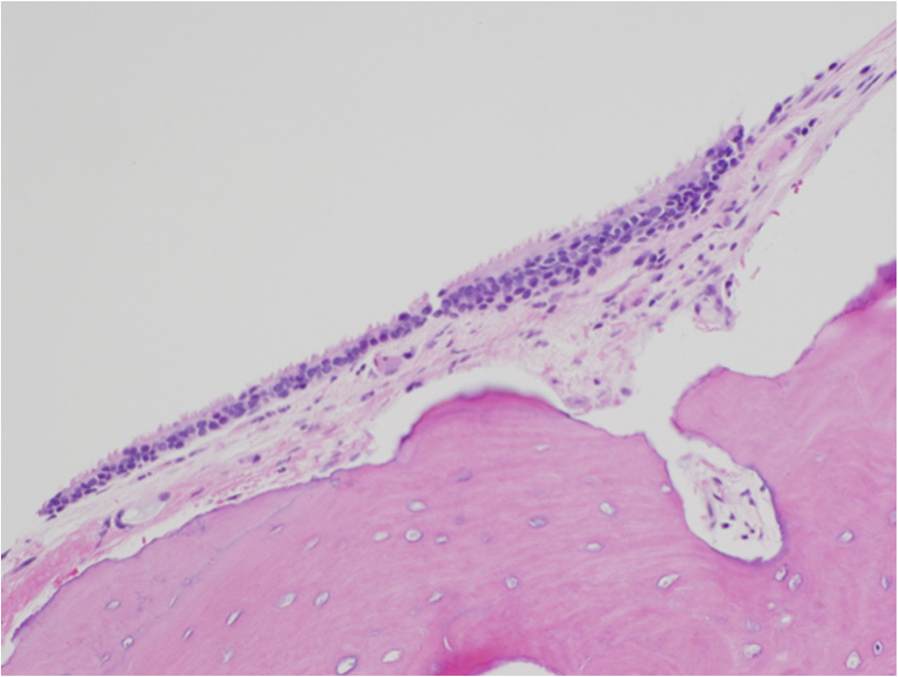
Histopathological section of middle ear osteoma showing the bone covering of cuboidal ciliated epithelium with a thin band of intervening fibrous tissue (stained with hematoxylin and eosin).

A 49-year-old Caucasian woman presented with progressive right-sided facial nerve weakness, slow in onset, and worsening over the past 2 years. At rest, she had normal symmetry and tone but there was incomplete eye closure on forced movement (House–Brackmann grade IV). She also complained of right-sided hearing loss and intermittent tinnitus. Otherwise, she was healthy and there was no mention of any vertigo or dizziness. No other focal neurological signs or symptoms were elicited. Otolaryngology referral was made due to the presence of dominant otologic symptoms, and she was placed on eye protective regimen. This included natural tears, ocular ointment, and eye patch at nighttime.Her head and neck and vestibular examinations were normal, other than a subtle right middle ear bony prominence noted on otoscopy. An audiogram revealed right mild conductive low-frequency hearing loss. A computed tomographic (CT) image of the temporal bone showed a hyperdense lesion in the right middle ear space (Figures 1 and 2). Middle ear exploration was then performed, which revealed an osseous lesion abutting the floor of the middle ear. Specifically, the lesion was found to involve the stapes and the area of the tympanic portion of her facial nerve, which runs above the footplate of the stapes. This was excised and the specimen was sent for histopathological examination (Figures 3 and 4), which resulted in a diagnosis of a middle ear osteoma. The tympanic portion of her facial nerve was decompressed at the same time.

After the operation she had partial recovery of her facial nerve function. More specifically, her facial nerve function improved from House–Brackmann grade IV to grade II at the 12-month follow-up visit. No recurrence was observed with prolonged follow-up of 5 years.

## Discussion

Temporal bone osteomas are rarely encountered benign neoplasms resulting from lamellar bone deposition most commonly in the external auditory canal [1,2]. Typically, these tumors appear as solitary, unilateral, and pedunculated lesions located in the lateral bony ear canal [3]. Osteomas can be differentiated from exostoses, since the latter usually presents as multiple, bilateral, and broad-based elevations of the medial bony external auditory canal [4].

Only one other case of middle ear osteoma presenting with facial nerve weakness is found in the literature [5]. In most cases, the confirmation of a diagnosis requires CT imaging, along with visual inspection during surgical exploration with histopathological analysis of the biopsied or excised specimen [6].

A review of the literature of middle ear osteoma cases revealed a male preponderance (2:1) with a median age at diagnosis of 28 years (mean 28.5 years; range, 5 to 27) [2]. The first report cases involved a pair of siblings and thus a genetic etiology was suggested [7]. However other possible causes, such as chronic inflammation due to exudative otitis, have also been proposed and the precise etiology of middle ear osteoma has yet to be clarified.

On histopathological examination, osteomas of the middle ear resemble those of the external auditory canal and can generally be characterized by the benign proliferation of cancellous bone [7]. They exhibit an abundance of fibrovascular channels surrounded by lamellar bone, which contains few osteocytes or lacunae (Figure 3) [4,8]. Focally, osteomas are usually covered by cuboidal ciliated epithelium with a thin band of intervening fibrous tissue (Figure 4).

Given that external auditory canal osteomas have a tendency for very slow growth and many cases are not associated with any significant clinical problems, some authors suggest long-term monitoring as a viable management option [7]. Yet, most middle ear osteomas present with an associated feature, such as conductive hearing loss and tinnitus, and therefore surgical excision is more readily applied to these lesions [2,7]. Further, middle ear osteomas can irreversibly injure the facial nerve and erode into the inner ear, causing vertigo and sensorineural hearing loss [5]. Hence, surgical excision to prevent these severe complications may be warranted. Within the middle ear, promontory is the most commonly involved site, followed by incus, pyramidal process, and the anterolateral wall of the epitympanum [2].

Other middle ear lesions that may present similarly include fenestral otospongiosis, ossifying hemangioma, osteoid osteoma, benign osteoblastoma, ossifying fibroma, fibrous dysplasia, osteochondroma, chondroma, calcified meningioma, isolated eosinophilic granuloma, giant cell tumor, and malignant masses, such as osteosarcoma and osteoblastic metastasis [2].

Our patient presented with a progressive facial nerve weakness, in addition to conductive hearing loss and tinnitus. This was the direct result of the osteoma encasing the stapes and compressing the tympanic portion of her facial nerve. Subsequently, surgical removal of the osteoma, along with the stapes, was performed. Her facial nerve was also decompressed at the same time. This has resulted in partial recovery of her facial nerve function (House–Brackmann grade II). Central lesions affecting the facial nerve, such as cerebellopontine angle tumors, can also present similar to our case [9]. Early recognition, referral, and treatment may also prevent permanent facial nerve dysfunction in these cases [9].

## Conclusions

Common causes of facial nerve weakness include cerebrovascular accidents, cerebellopontine angle tumors, and Bell’s palsy. Very rarely, middle ear tumors present with facial nerve dysfunction. The weakness is typically due to a compressive effect on the middle ear portion of the facial nerve. Early recognition is crucial since removal of these lesions may lead to the recuperation of facial nerve function.

## Consent

Written informed consent was obtained from the patient for publication of this case report and any accompanying images. A copy of the written consent is available for review by the Editor-in-Chief of this journal.

## Abbreviations

CT: Computed tomography.

## Competing interests

The authors declare that they have no competing interests.

## Authors’ contributions

CKC analyzed and interpreted the patient data. MB analyzed and interpreted the patient data and performed the surgery. MC analyzed and interpreted the patient data. PH analyzed and interpreted the patient data. All authors read and approved the final manuscript.
